# Supplementary figures and images for: Identification of exosomal miRNAs associated with the anthracycline-induced liver injury in postoperative breast cancer patients by small RNA sequencing
Source: PeerJ. 2020 Apr 24;8:e9021. doi: 10.7717/peerj.9021 (PMC7185038; doi:10.7717/peerj.9021)

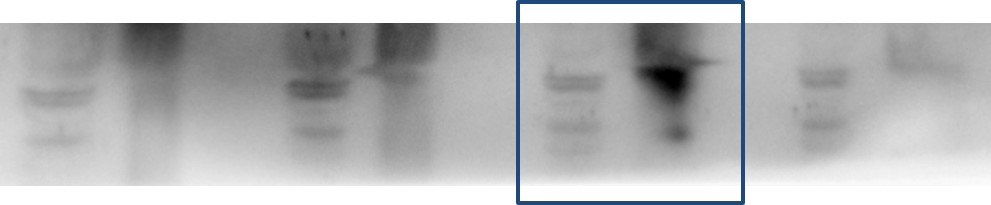

Supplement: Data S2 [file peerj-08-9021-s004.zip › Figure 2C CD63.jpg]

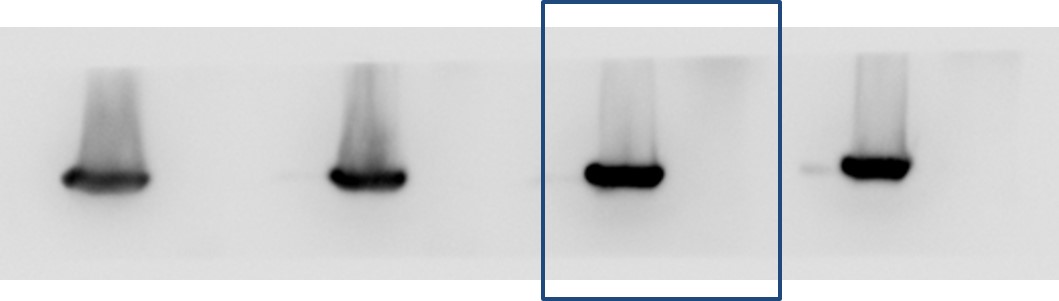

Supplement: Data S2 [file peerj-08-9021-s004.zip › Figure 2C Calnexin.jpg]

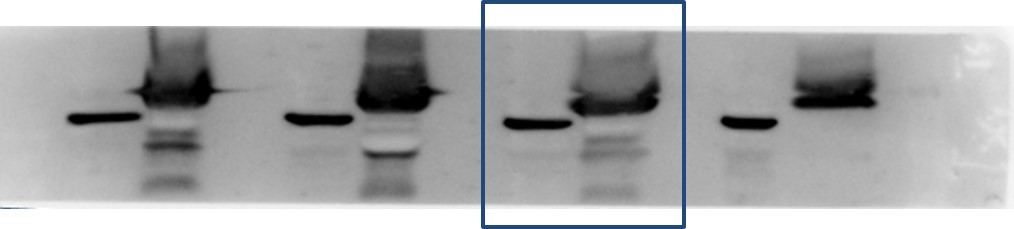

Supplement: Data S2 [file peerj-08-9021-s004.zip › Figure 2C Tsg101.jpg]

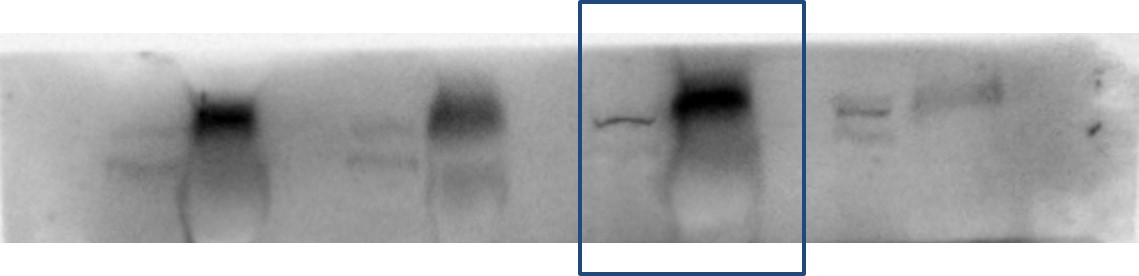

Supplement: Data S2 [file peerj-08-9021-s004.zip › Figure 2C Alix.jpg]
